# Supplementary material for: Sonoluminescence Spectra in the First Tens of Seconds of Sonolysis of [BEPip][NTf2], at 20 kHz under Ar
Source: Molecules. 2022 Sep 16;27(18):6050. doi: 10.3390/molecules27186050 (PMC9502986; doi:10.3390/molecules27186050)
Supplement: Supplementary file 1 [file molecules-27-06050-s001.zip › molecules-1885572-supplementary.pdf]

molecules

Sonoluminescence Spectra in the First Tens of Seconds of Sonolysis of [BEPip][NTf2], at 20 kHz under Ar

Rachel Pflieger [1,*], Manuel Lejeune [2] and Micheline Draye [2]

[1] ICSM, Univ Montpellier, CEA, CNRS, ENSCM, F-30207 Bagnols-sur-Cèze, France
[2] EDYTEM, University of Savoie Mont Blanc, F-73000 Chambéry, France
* Correspondence: rachel.pflieger@cea.fr

Supporting Information

Citation: Pflieger, R.; Lejeune, M.; Draye, M. Sonoluminescence Spectra in the First Tens of Seconds of Sonolysis of [BEPip][NTf2], at 20 kHz Under Ar. *Molecules* **2022**, *27*, 6050. https://doi.org/10.3390/molecules27186050

Academic Editor: Alessandro Chiasera

Received: 10 August 2022
Accepted: 6 September 2022
Published: 16 September 2022

Publisher's Note: MDPI stays neutral with regard to jurisdictional claims in published maps and institutional affiliations.

Copyright: © 2022 by the authors. Submitted for possible open access publication under the terms and conditions of the Creative Commons Attribution (CC BY) license (https://creativecommons.org/licenses/by/4.0/).

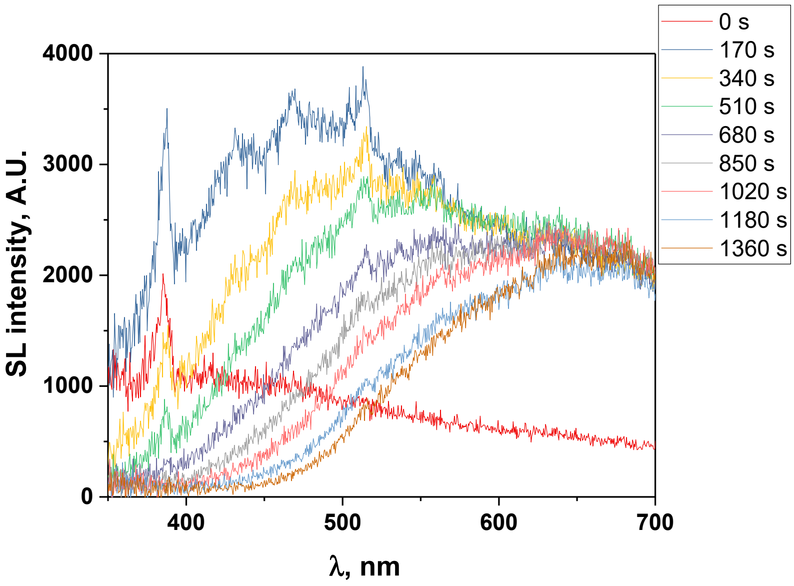

Figure S1. Time-evolution of the SL spectra of water-saturated [BEPip][NTf2] sonicated at 20 kHz under Ar flow. Degradation of the IL leads to increasing absorption of the SL light.

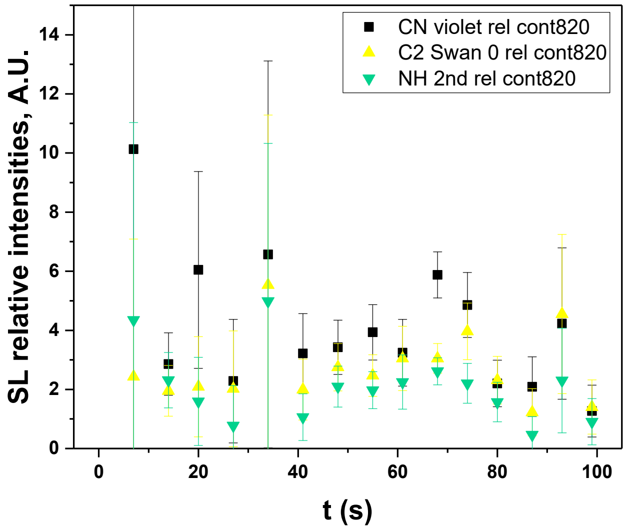

Figure S2. Evolution of the intensity of molecular emissions relatively to the SL continuum intensity (dry [BEPip][NTf_2]) – the fact that they are not constant indicates a non perfect proportionality between the number of SL emitting bubbles and the number of bubbles presenting droplet injection.

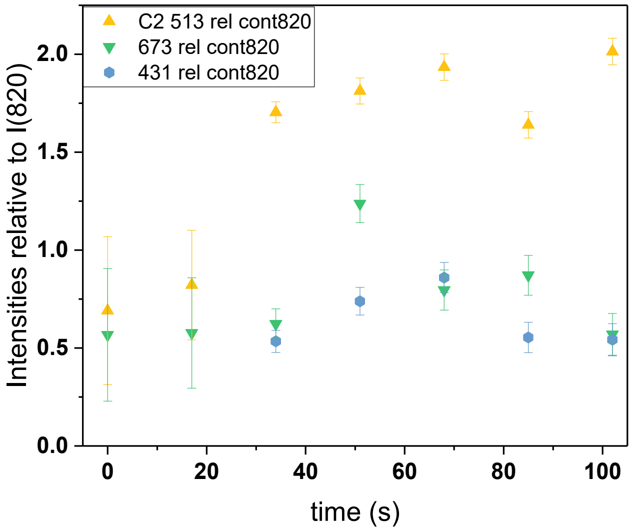

Figure S3. Evolution of the intensity of molecular emissions relatively to the SL continuum intensity (water-saturated [BEPip][NTf_2]).
